# Organic bromine compounds produced in sea ice in Antarctic winter

**DOI:** 10.1038/s41467-018-07062-8

**Published:** 2018-12-11

**Authors:** Katarina Abrahamsson, Anna Granfors, Martin Ahnoff, Carlos A. Cuevas, Alfonso Saiz-Lopez

**Affiliations:** 1 Department of Marine Sciences, University of Gothenburg, Carl Skottbergs gata 22B, SE-41319 Gothenburg, Sweden; 2 AstraZeneca, Product Technology and Development, SE-43183 Mölndal, Sweden; 3 Department of Atmospheric Chemistry and Climate, Institute of Physical Chemistry Rocasolano, Serrano 119, 28006 Madrid, Spain

## Abstract

During polar springtime, active bromine drives ozone, a greenhouse gas, to near-zero levels. Bromine production and emission in the polar regions have so far been assumed to require sunlight. Here, we report measurements of bromocarbons in sea ice, snow, and air during the Antarctic winter that reveal an unexpected new source of organic bromine to the atmosphere during periods of no sunlight. The results show that Antarctic winter sea ice provides 10 times more bromocarbons to the atmosphere than Southern Ocean waters, and substantially more than summer sea ice. The inclusion of these measurements in a global climate model indicates that the emitted bromocarbons will disperse throughout the troposphere in the southern hemisphere and through photochemical degradation to bromine atoms, contribute ~ 10% to the tropospheric reactive bromine budget. Combined together, our results suggest that winter sea ice could potentially be an important source of atmospheric bromine with implications for atmospheric chemistry and climate at a hemispheric scale.

## Abstract (web summary)

Short-lived natural bromocarbons, which contribute to ozone depletion in the atmosphere, are believed to be produced through light-driven processes, mainly in oceans. Here the authors present bromocarbon measurements in snow, sea ice, and air during polar winter that show an unexpected source of bromine to the polar atmosphere during periods of no sunlight.

## Introduction

Bromine-containing organic compounds (bromocarbons) are ubiquitous in the oceans, and they are mainly formed by macro- and microalgae^1^. Of these naturally produced substances, bromoform (CHBr_3_) is the best known, since it is the most abundant brominated organic. It is formed in enzymatic processes developed to protect algal cells of reactive oxygen species, such as hydrogen peroxide, formed during photosynthesis^1,2^. In addition, several other bromocarbons are formed, such as dibromomethane (CH_2_Br_2_), dibromochloromethane (CHBr_2_Cl), and bromodichloromethane (CHBrCl_2_), either directly through the same enzymatic pathway, or by nucleophilic substitution of CHBr_3_. In the atmosphere, bromocarbons are photochemically degraded to reactive bromine which initiate the depletion of ozone and mercury, a contaminant of global concern^3–5^. BrO, formed by the reaction of atmospheric Br atoms with O_3_, has an impact on the sulfur cycle through the oxidation of dimethyl sulfide (DMS), leading to a 18% reduction in the DMS burden and lifetime^6^. The Br atom is the dominant oxidizer in mercury depletion events in the polar atmosphere^7^. Reactive bromine from the decomposition of bromocarbons also contributes to the depletion of ozone in the lower stratosphere^8^. The interplay between the oceanic sources of bromocarbons and their atmospheric impacts has led to the suggestion that these compounds are a link between climate change and atmospheric ozone^9^.

Recently, it has been shown that variability in polar tropospheric bromine oxide (BrO) levels is correlated with first year sea ice concentrations, through the production of halogens in sea ice regions^10^. Reactive bromine is anti-correlated with ozone, which suggests that the efficiency of halogen-driven polar tropospheric ozone depletion is related to sea ice dynamics and biogeochemical processes. Research on the role of sea ice in the formation of reactive halogens has in recent years mainly focused on the inorganic chemistry of saline surfaces, where sea-salt halides are oxidized to more reactive forms^11,12^. Similarly, inorganic bromine has also been shown to form in saline snow^13^. Arctic and Antarctic sea ice algae have been estimated to emit organo-bromine with ~ 70–80 × 10^8^ g yr^−1^, suggesting that the produced compounds are released into seawater and then reach the atmosphere^14^. Other investigations have pointed out the possibility of sea ice as a source of atmospheric CHBr_3_ in the Amundsen Sea (Antarctica) during the sunlit period^15^. In coastal Antarctica, concentrations of iodo-carbons in sea ice were found to be strongly enhanced relative to seawater, suggesting a significant local source within the sea ice^16,17^. Therefore, although halocarbons are thought to be produced within sea ice, two key questions remain unknown: (i) whether this source is significant in supplying bromocarbons to the atmosphere relative to the flux from the surface waters in leads and the open ocean, and (ii) the role of biogeochemical cycles of bromocarbons during polar winter, as their production, and thereby emissions, have so far been assumed to require sunlight.

In this work, we report profile measurements of bromocarbons in snow, sea ice, and air during Antarctic winter that represent a new source of atmospheric bromine during the polar night. We combine the observations with a state-of-the-art global chemistry-climate model. The results provide strong evidence that Antarctic winter sea ice is a significant source of bromocarbons, which spread and contribute to the burden of atmospheric bromine throughout the southern hemisphere.

## Results

### Bromocarbon concentrations in Antarctic winter

**Fig. 1:**
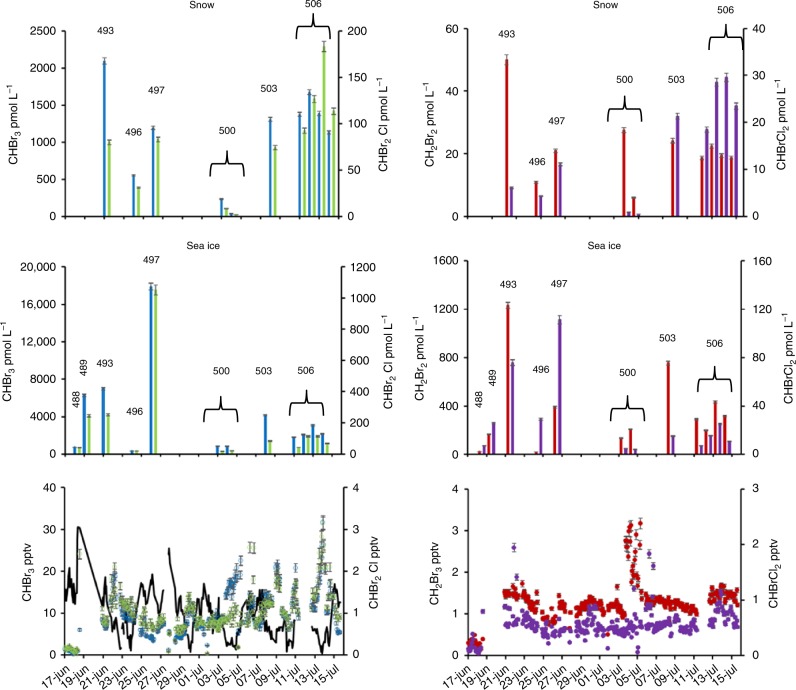
Time series of sea ice, snow, and air measurements of bromocarbons during the Antarctic winter in 2013. From top to bottom: snow, sea ice and air measurements of CHBr_3_ (blue), CHBr_2_Cl (green), CH_2_Br_2_ (red), and CHBrCl_2_ (purple). Wind speed (black line) is shown in bottom panel. At stations 496, 500, and 506 duplicate ice cores were sampled, and the average values are presented. In total, 23 ice cores and 30 snow samples were analyzed. Time resolution of measurements in air was 40 min. For geographical location of stations see Supplementary Table 1 and Supplementary Figure 1. The error bars relates to the instrumental errors

A field campaign was performed from 8 June to 12 August 2013 in the Weddell Sea, Antarctica (Supplementary Figure 1). Here we report data from the dark period of the cruise 17 June to the 15 July. The concentrations of bromocarbons (CHBr_3_, CH_2_Br_2_, CHBr_2_Cl, and CHBrCl_2_) were measured in snow, sea ice, and air to estimate their source strength of seasonal sea ice in the absence of sunlight (Fig. 1). These measurements were made at nine stations where 23 ice cores were collected. The thickness of the first year sea ice over which measurements were made varied between 43 and 118 cm, whereas the snow thickness varied between zero and 5–40 cm (Supplementary Table 1).

The air measurements (located at 20 m above sea ice level) revealed unexpectedly high concentrations of bromocarbons under conditions of solar zenith angle (SZA) larger than 90° (i.e., full darkness), with average values of 9.5 pptv (CHBr_3_), 0.53 pptv (CHBrCl_2_), 0.91 pptv (CHBr_2_Cl), and 1.2 pptv for CH_2_Br_2_ (Fig. 1). As a comparison, studies of bromocarbons in air in the Arctic region before polar sunrise measured concentrations of CHBr_3_, CHBrCl_2_, CHBr_2_Cl, and CH_2_Br_2_ to be 2.6, not detected, 0.3 and 0.8 pptv, respectively. After sunrise, the concentrations dropped by half for CHBr_3_, whereas the concentrations remained fairly constant for the other compounds^18^. Compared with observations made in the summertime in the Southern Ocean^15,19^, the average winter air concentration of bromoform was *ca*. 10 times higher. Similarly, the maximum concentration of CH_2_Br_2_ found in winter was 2.9 pptv compared with 1.4 during summer and that of CHBr_2_Cl was 2.5 pptv in summer^19^. The boundary layer in Antarctica during winter is much lower than over open ocean^20^, therefore these bromocarbon fluxes could lead to lower air concentrations under higher boundary layer height conditions. The variations in concentration were not related to wind direction. Backward air mass trajectories revealed that the dominant wind direction was from West and South (Supplementary Figure 1). We note that the highest air concentrations occurred at low wind speeds ( < 6 m s^−1^) (Fig. 1) associated with stable shallow boundary layers^20^.

**Fig. 2:**
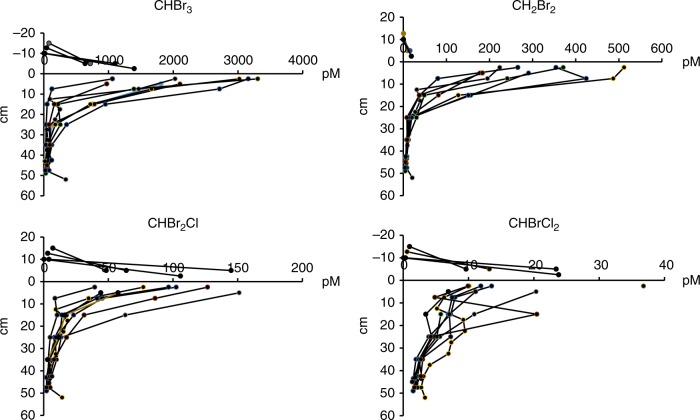
Profiles of brominated halocarbons (CHBr_3_, CH_2_Br_2_, CHBr_2_Cl, and CHBrCl_2_) in sea ice and snow. Data from station 506 include eight sea ice core profiles and four snow profiles. The line at 0 cm indicates the sea ice–snow interface. The total snow depth was 17 cm. The complete concentration data set is found in Supplementary Tables 3–5

In addition to the air observations, sharp concentration gradients in the vertical profiles of bromocarbons in sea ice and snow were observed, with highest values at the ice–snow interface (Fig. 2, Supplementary Figure 2). This implies that the observed bromocarbons were formed at the interface and diffused out of the snow. Sharp concentration gradients have previously been observed in Arctic sea ice during spring with similar distribution patterns in sea ice^21^ and snow^22^. Note that on one location (Station 500) one of the coring sites was flooded, which led to slightly different profiles, as indicated by the high salinity found close to the sea ice interface (Supplementary Figure 2, Supplementary Table 2–3). The highest concentrations found at the interface during the Antarctic winter were 1200, 1100, 270, and 18000 pM for CH_2_Br_2_, CHBr_2_Cl, CHBrCl_2_, and CHBr_3_, respectively (Fig. 1), which can be compared with summer sea ice maximum values of 90, 14, 42, and 420 pM, respectively^23^. During summer, the depth profiles differed with bromocarbon concentrations varying to a large extent with chlorophyll-a^23^.The complete set of air, snow, and sea ice concentrations for all stations, salinity, temperature, and brine volume data are given in Supplementary Tables 2–4. Together with the air observations, the measured profiles across snow and sea ice show that bromocarbons are produced locally in the Antarctic winter sea ice.

## Discussion

We now turn to the mechanism behind the observed production of bromocarbons in winter sea ice. The natural formation of bromocarbons has previously been demonstrated to be light dependent^2^, and include enzymatic pathways that rid cells of reactive oxygen species formed mainly during photosynthesis. The reduction of hydrogen peroxide by haloperoxidases results in the formation of hypohalous acids, such as HOBr, which subsequently reacts with dissolved organic matter, forming bromocarbons^24^. Laboratory experiments have shown that there is an abiotic pathway for HOBr production through the reaction of ozone with bromide that occurs in darkness^25^. This proceeds via ozone deposition to the ice surface followed by oxidation of bromide. On ice surfaces, a disordered layer of molecules exist, which has been shown to favor halogen interactions. Also, the freezing of seawater enhances the ionic strength that leads to enhanced rates of chemical reactions^26^. Recent investigations have shown that the oxidation of bromide by ozone involves the formation of the intermediate ozonide Br·OOO^−^ in aqueous bulk solution with a preference for the liquid–gas interface, and the authors conclude that the oxidation of bromide on surfaces plays a more important role than previously thought^27^.

Subsequently, a number of different organic compounds such as ketones, phenols, alkenes etc., can react with HOBr to form bromocarbons^28^. Therefore, the differences in organo-bromine concentrations found in our ice cores can be due to differences in composition of the organic compounds being brominated. The similarities in depth profiles within the sea ice between the individual bromocarbon indicate similar formation mechanisms for all four investigated bromocarbons.

Micro-organisms could also be responsible for the bromocarbon production, as they have been shown to be active in sea ice during winter^29,30^. For instance, if the enzymatic pathways are active, the stress caused by the measured high salinity at the ice interface, low temperatures, and/or inclusion in the ice lattice could increase the production, and contribute to the high concentrations of bromocarbons found at the snow/ice interface. Hence, although little is known about how bromocarbons form under darkness; our results provide a strong case, for the first time, that first year Antarctic sea ice has extremely high bromocarbon concentrations at a period with no sunlight.

Next, the magnitude of the release of individual bromocarbons from the ice surface to the atmosphere was calculated based on the assumption that the bromocarbons were formed on the surface of sea ice and subsequently diffused through the snow to the atmosphere. The interstitial air concentrations were calculated from the concentrations found in melted snow, the fraction of water and air, respectively, and the Henry's law constants (Methods). We found the concentration gradient in snow to decrease linearly (Fig. 2, Supplementary Table 4). Adsorption and desorption studies of halocarbons (i.e., CHCl_3_ and CHBr_3_) on ice revealed that CHBr_3_ is mobile at temperatures as low as 85 K, which allows us to infer that the bromocarbons were not adsorbed/desorbed on snow surfaces.

It has been shown that wind pumping could be important for the flux of gases through the snow. In such cases, concentration gradients are absent and the fluxes are not governed by diffusive processes^31^. As our results show the presence of concentration gradients that could be assigned to diffusive processes, a simplified diffusion calculation was performed using the molecular diffusivities in air (Methods). These diffusivities are comparable to the effective diffusivities calculated for SF_6_ in snow at Summit, Greenland^32^. The median ice–snow–air fluxes of individual halocarbons varied between 1 and 50 nmol m^−2^ h^−1^ (Table 1). We performed a one sample Wilcoxon signed rank test that shows that the median “fluxes” are higher than 16 nmol m^−2^ h^−1^ (CHBr_3_); 0.5 nmol m^−2^ h^−1^ (CH_2_Br_2_); 0.3 nmol m^−2^ h^−1^ (CHBrCl_2_ and 0.5 nmol m^−2^ h^−1^ (CHBr_2_Cl) at a significance level of 0.05. Fluxes from all stations are given in Supplementary Table 5. The total error of the individual fluxes was estimated to be 13% based on the measurement uncertainties in concentrations and in the snow depth measurements.

**Table 1:** Estimated flux of bromocarbons from sea ice

	Flux (nmol m^−2^ h^−1^) stations without snow	Flux (nmol m^−2^ h^−1^) stations with snow (*n* = 9)
CH_2_Br_2_		
Range	20; 50	0.5–7
Median		2
CHBrCl_2_		
Range	3; 5	0.1–8
Median		1
CHBr_2_Cl		
Range	50; 70	0.5–8
median		2
CHBr_3_		
Range	800; 1700	8–200
Median		50

The fluxes were calculated utilizing the diffusion through snow calculations

^a^ Stations 488 and 489 had no snow cover

^b^ Station 493–506 had a snow cover of 5–40 cm

On board, attempts were made to perform an air gradient flux estimate. There were some indications that the air concentration just above the snow had elevated concentrations compared with a height of 2.2 or 20 m (Supplementary Figure 3). However, the precision of our analytical method was not good enough to use the sometimes small differences in concentrations for flux calculations. Also, there was no possibility to take into consideration the influence of the ship on the air movement. Eddy covariance or air gradient flux measurements depend on the capability of rapid measurements at relevant concentrations. In our case, the concentrations are low (0.5–30 pptv) and required sample pre-treatment. The variations in concentrations during the above described measurements are exemplified in Supplementary Figure 3. At station 506 the concentrations of CHBr_3_ varied between 20 and 100 pptv with the highest values at midnight. These variations were related to wind speed. Around midnight the wind speed dropped from ca 7 m s^−1^ to ca 1 m s^−1^ to, later in the night, increase again to ca 9 m s^−1^. This trend was valid for all bromocarbons (positive linear regression between bromocarbons with *R*^2^ values of 0.7).

**Fig. 3:**
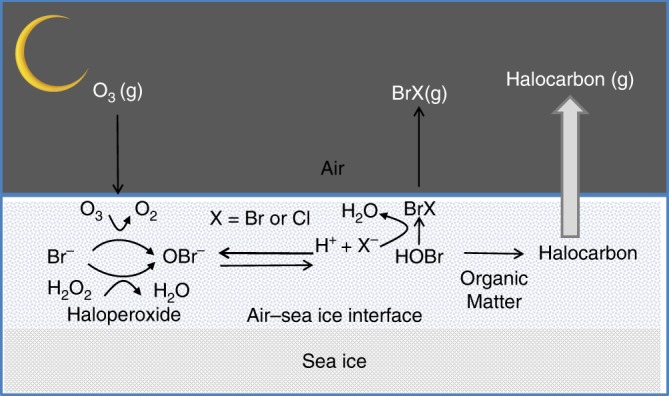
Schematic of the possible release mechanisms of bromocarbons from sea ice in the Antarctic winter

As no earlier attempts have been made to estimate the flux of bromocarbons from sea ice surfaces through snow to the atmosphere we here performed calculations using an established method for calculating sea-air fluxes, utilizing the upper most sea ice values instead of seawater concentrations and the air concentrations measured in the snow closest to the ice surface. For stations 488 and 489 that did not have any snow cover (Table 1) we used the measured wind speed. We found that the two methods (Methods) produced median values of flux estimates in the same order of magnitude at the stations where multiple cores were sampled (Supplementary Table 5). This fact could be seen as an indirect evidence that the flux through snow could be sustained by the sea ice surface. In Fig. 1, the snow concentrations could be compared with the upper most layer of the sea ice. In general, the higher the concentration in sea ice the higher the values in snow (Supplementary Tables 2–3, Fig. 2 and Supplementary Figure 2). A principal component analysis (Supplementary Figure 4) also reveals that the highest concentrations of bromocarbons were found in the upper most part of the core and in the snow closest to the ice. The variability in sea ice is known to be one major obstacle for extrapolating physical, chemical, and biological properties. On kilometer scales, it can be concluded that the distribution pattern was consistent in the seasonal ice. However, there was a large variation in the concentrations of individual bromocarbons in snow and surface sea ice, which was reflected in the calculated fluxes which varied with one order of magnitude. The variability on meter scales showed less variation (Supplementary Table 5). The snow concentrations were clearly linked to the surface concentrations in sea ice (Fig.1), and thereby the magnitude of the flux. At station 500, where parts of the flow were flooded, the lowest concentrations were found and also the lowest flux. Predicted global sea to air fluxes of CHBr_3_ and CH_2_Br_2_ in the Southern Ocean are lower than our ice–snow–air fluxes by several orders of magnitude^33^. This demonstrates that areas covered with seasonal ice are potential foci for bromocarbon emissions to the atmosphere, even in the absence of sunlight. Figure 3 depicts a simplified schematic of the ice–snow–air interface and the suggested release mechanisms of organic bromine from Antarctic winter sea ice to the atmosphere.

**Fig. 4:**
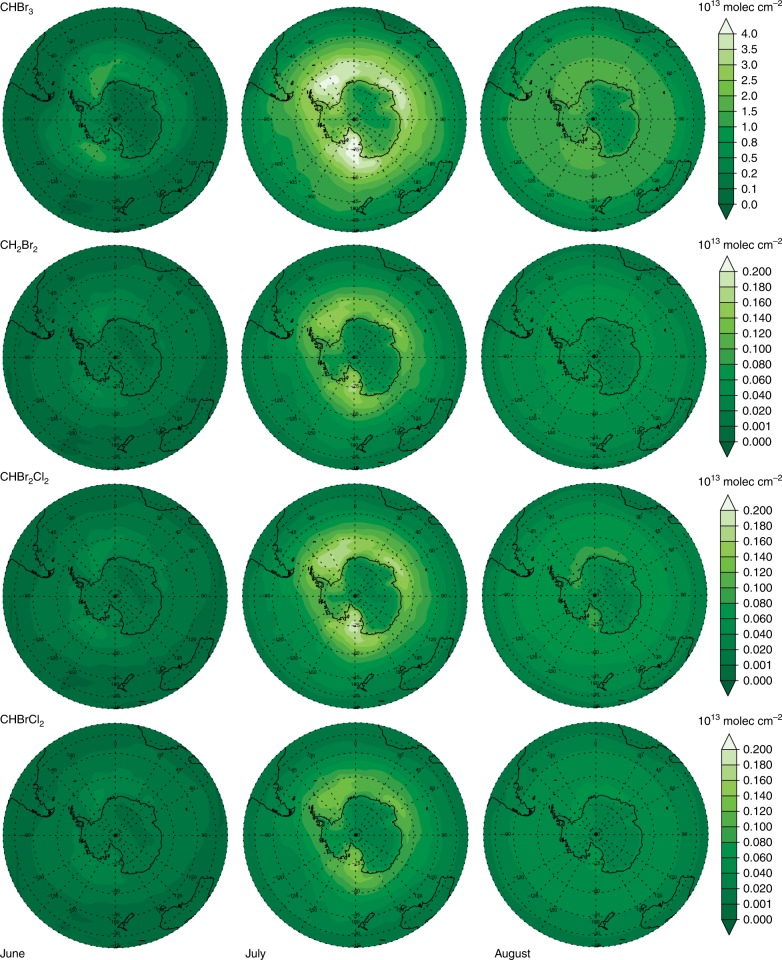
Modeled distribution of monthly averaged tropospheric (CHBr_3_, CH_2_Br_2_, CHBr_2_Cl, and CHBrCl_2_) over the Southern Hemisphere. This spatial distribution is the result of sea ice emissions of bromocarbons during the Antarctic winter using the averaged fluxes of bromocarbons from Table 1 (flux in stations with snow)

We now use a state-of-the-art global chemistry-climate model^34,35^ constrained with the observed bromocarbons fluxes during the period of measurements and over Antarctic first year sea ice (Methods). The results show an accumulation of brominated halocarbons in the atmosphere during the polar winter until the austral sunrise in August when photodecomposition starts to reduce the bromocarbon concentrations (Fig. 4, Supplementary Figures 5, 6, 7). Remarkably, since bromocarbons are relatively long-lived (weeks to months) the winter sea ice emission combined with atmospheric transport results in the distribution of these species throughout the troposphere over the entire southern hemisphere (Fig. 4). The model driven by the observed ice–snow atmosphere bromocarbon fluxes satisfactorily simulates the range of measured surface air concentrations of organo-bromine species (Table 2 and Supplementary Figure 8).

**Table 2:** Measured and modeled surface air bromocarbon concentrations

Bromocarbon species	Measured range/pptv	Modeled range/pptv
CHBr_3_	nd–32	0.2–40
CHBr_2_Cl	0.1–3.2	0.1–1.8
CH_2_Br_2_	0.3–3.2	0.9–2.3
CHBrCl_2_	0.1–1.9	0.1–1.4

The modeled values correspond to a layer 0–200 m. Altitude for the same date and location of the measurements

In the austral spring, the arrival of sunlight initiates the photochemical breakdown of bromocarbons, accumulated during the polar night, leading to the formation of reactive inorganic bromine species throughout the troposphere of the southern hemisphere (Supplementary Figures 9, 10, 11). Increases of 1 pptv of active bromine are simulated up to the mid- to upper-troposphere over large regions of the southern hemisphere, far away from the Antarctic sea ice source region, i.e., 80°–30°S (Supplementary Figure 9). We calculated that integrated over the southern hemisphere, the polar night emission of organic bromine during the period of measurements results in an increase of ~ 10% of tropospheric reactive bromine (Br, Br_2_, BrO, HBr, HOBr, BrONO_2_, BrCl)^36^, based on the average emission fluxes of bromocarbons from Table 1 (Flux in stations with snow). For the lower and upper limits of bromocarbon emission fluxes (Table 1), the increase in tropospheric reactive bromine is 1.5% and 31%, respectively. This can be considered a lower limit since a sustained emission during the whole polar night and sunlit periods (which are not currently considered in chemistry-climate models) could have larger hemispheric impacts on the levels of reactive bromine in the atmosphere.

A final point to consider is the geographical scale of emissions. The Antarctic sea ice cover in winter is ~ 19 × 10^6^ km^2^. If we use this area in combination with the range of fluxes given in Table 1, the flux of bromocarbons to the atmosphere would range from of 0.6–1.8 Gmol Br per month of polar night, which should be compared with the estimated global contribution of 2.4–3.5 Gmol Br y^−1^ based on CHBr_3_ and CH_2_Br_2_^33^. The model calculations indicate that the winter emissions of bromocarbons spread throughout the southern hemisphere, and due to their relatively long lifetime they could even be transported to the stratosphere.

In summary, this study underscores that a large source of bromine from winter sea ice is currently neglected in climate models. The relatively long atmospheric lifetime of Antarctic winter sea ice-emitted bromocarbons allows their transport to lower latitudes and even to the stratosphere, adding to the global atmospheric bromine burden. Consideration of this new source of polar bromine requires re-assessment of the bromine-mediated impacts on the global tropospheric ozone budget and mercury deposition.

## Methods

### Halocarbon measurements

Data were collected during the ANTXXIX/6 expedition conducted during austral winter aboard the R.V Polarstern from 8 June to 12 August 2013 in the Weddell Sea (Supplementary Figure 1). Samples of ice, snow, and brine, were collected at nine different stations, during the dark period of the cruise (Supplementary Table 1). At stations 500 and 506, multiple samples were collected over two or more days. A Mark II coring system from Kovacs Enterprise with a diameter of 0.09 m made out of a light weight filament wound composite tube with plastic fitting was used. Ice cores were divided into 10 cm or 5 cm sections and individually packed in gas-tight Tedlar^TM^ bags. The air surrounding the sample was removed from the bags using a manual pump according to Granfors et al.^23^. The ice samples were thawed in darkness at room temperature for ~ 24 h. Snow samples were collected as close as possible to the ice coring site (maximum distance ca 10 m) and divided into 5–10 cm sections, individually packed in gas-tight Tedlar© bags. The snow samples were thawed in darkness at room temperature for ~ 12 h. The halocarbon compounds CHBr_3_, CH_2_Br_2_, CHCl_2_Br, and CHClBr_2_ were quantified. They were pre-concentrated using three purge-and-trap systems: Velocity XPT (Teledyne Tekmar) connected to an autosampler (AQUATek70, Teledyne Tekmar), and two custom-made purge-and-trap system, which were coupled to gas chromatographs with electron capture detection (Varian 3800), according to the methods described by Mattsson et al.^15^. One of the custom-made purge-and-trap instruments (custom-made system 1 Supplementary Table 6) was equipped for air sample analysis in addition to water sample analysis. Air was continuously drawn through a 100 m, 4 mm inner diameter, Teflon tube by an air pump located downstream from the sampling loop. This instrument was also fed with a continuous stream of water from the ship's surface water inlet. The system was set up to automatically alternate between water and air sampling. The systems were calibrated with external standards of CH_2_Br_2_ (Merck, 99%), CHBrCl_2_ (Fluka, > 98%), CH_2_ClI (Fluka, > 97%), CHBr_2_Cl (Fluka, > 97%), and CHBr_3_ (Merck, > 98%) diluted from stock solutions in methanol (Sigma-Aldrich, suitable for purge-and trap analysis) in seawater to give final concentrations in the picomolar range in the purge chamber. The same standard solutions were used for calibration of air measurements. The detection limits of the systems are given in Supplementary Table 6. The overall repeatability for individual compounds was between 1% and 5%. The three instruments were inter-calibrated using standards as well as samples with high and low concentrations of the compounds.

Sea ice temperatures were measured immediately after the ice core was recovered at 10 cm intervals using a digital thermistor, Amadigit, resolution 0.1 °C (summer expedition) and a high precision RTD thermometer with a Pt100 temperature sensor with an accuracy of ± 0.1 °C (winter expedition). Salinity and conductivity of the melted sea ice were measured using a conductivity meter, WTWCond330i or 3210, with a precision and accuracy of ± 0.1.

Brine volume (*v*_*b*_) was derived from ice temperature (*T*_i_) and bulk salinity (*S*_i_) according to the equation of Frankenstein and Garner [1967]^37^ (Eq. 1).

1 $$v_{\mathrm{b}} = S_{\mathrm{i}}\left( {0.0532 - 4.919/T_{\mathrm{i}}} \right)$$

For presentation of the vertical distribution of halocarbons in sea ice, halocarbon concentrations in bulk ice were divided by the brine volume calculated for each sample. This brine normalized concentration represents the estimated concentration of halocarbons in the sea ice brine.

### Flux calculations

Stations 493–506 had a snow cover of 5–40 cm. Vertical profiles in snow (thickness > 10 cm) indicated that the snow was ventilated above this level. The interstitial air concentrations were calculated from the measured concentrations in melted snow, the volume of air and water in the samples and the Henry's law constant at ambient temperature. The linearly decreasing concentrations in snow along with the following air diffusion coefficients were used to calculate fluxes according to Fick's first law (Eq. 2) (CHBr_3_ 0.0149; CH_2_Br_2_ 0.0287; CHBrCl_2_ 0.0298; CHBr_2_Cl 0.0196 cm^2^ s^−1^).

2 $${\mathrm{Flux}} = \Delta \left[ X \right]_{{\mathrm{air}}} \times D_{\mathrm{a}}{\mathrm{/}}z$$

where [*X*] is the individual concentration of bromocarbons, *D*_*a*_ the molecular diffusivity and *z* is the snow depth.

To compare, fluxes were also derived using a sea-air flux model according to Waninkoff and McGillis^38^. The Schmidt numbers were calculated based on brine salinities and ice surface temperatures^39^. Henry's law constants were calculated according to Moore et al.^40^. The air concentrations used in the calculations were those from the air measured in snow closest to the ice surface. This exercise yielded median fluxes of the bromocarbons CHBr_3_, CH_2_Br_2_, CHCl_2_Br, and CHClBr_2_ of 70, 15, 0.4, and 4 nmol m^−2^ h^−^respectively.

### The CAM-Chem model

The model employed in this work is the global 3-D chemistry-climate model CAM-Chem (Community Atmospheric Model with chemistry, version 4.0) to assess the impact of the measured bromocarbons emissions upon the distribution of bromocarbons and reactive bromine throughout the southern hemisphere. The model includes a comprehensive chemistry scheme to simulate the evolution of trace gases and aerosols in the troposphere and the stratosphere^34^. The model runs with the iodine and bromine chemistry schemes from previous studies^36,41–43^, including the photochemical breakdown of bromo- and iodo-carbons emitted from the oceans^35^ and abiotic oceanic sources of HOI and I_2_^44^. For this work, emissions of CHBr_3_, CH_2_Br_2_, CHBr_2_Cl, and CHBrCl_2_ from Antarctic first year sea ice at SZA higher than 90° have been implemented, according to measured fluxes. These emissions are only active during the campaign time period (18 June to 14 July), as a linear function of first year sea ice fraction. CAM-Chem has been configured in this work with a horizontal resolution of 1.9° latitude by 2.5° longitude and 26 vertical levels, from the surface to ∼ 40 km altitude. All model runs in this study were performed in the specified dynamics mode^34^ using offline meteorological fields instead of an online calculation, to allow direct comparisons between different simulations. This offline meteorology consists of a high-frequency meteorological input from a previous free running climatic simulation. The MERRA reanalysis data set^45^ is used in the model to supply the implemented sea ice fields.

## Electronic supplementary material

Supplementary Information

Description of Additional Supplementary Files

## Data Availability

The code and data that support the findings of this study are available upon request.
