# Supplementary material for: Organic bromine compounds produced in sea ice in Antarctic winter
Source: Nat Commun. 2018 Dec 11;9:5291. doi: 10.1038/s41467-018-07062-8 (PMC6290016; doi:10.1038/s41467-018-07062-8)
Supplement: Supplementary file 1 — Supplementary Information [file 41467_2018_7062_MOESM1_ESM.pdf]

Supplementary information

Organic bromine compounds produced in sea ice in Antarctic winter

Abrahamsson et al.

Supplementary Figure 1-11

Supplementary Tables 1-6

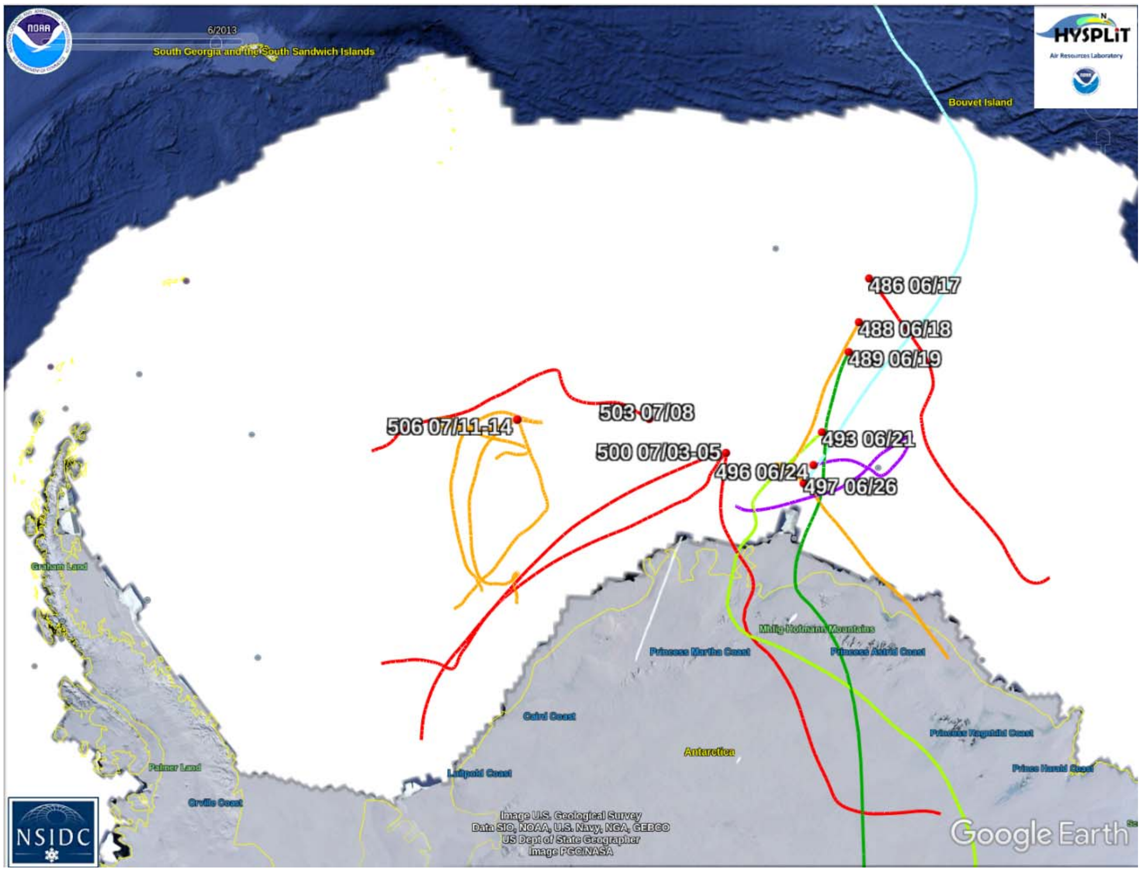

Supplementary Figure 1. **Location of Stations during the Expedition and backward air mass trajectories**. Stations occupied during the ANT XXIX/6 expedition to the Weddell Sea. Stations 486 to 506 are included in the results. For each station the backward air mass trajectories are shown, as calculated by the National Oceanic and Atmospheric Administration (NOAA) Hybrid Single Particle Lagrangian Integrated Trajectory Model (HYSPLIT), at 1 m above ground and three days before arriving time. Sea ice extent (averaged in June 2013) from National Snow and Ice Data Center. [NSIDC_SepSeaIceExtent.kmz] NSIDC data on Google Earth. Digital media. http://nsidc.org/data/google_earth. Accessed [2018/03/12].

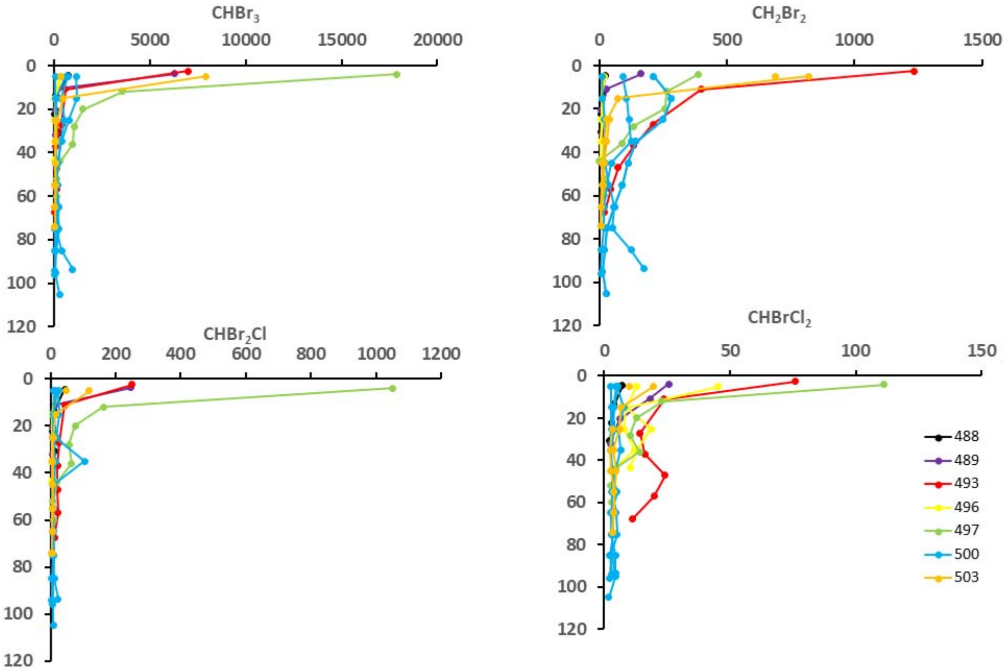

Supplementary Figure 2. **Depth profiles of bromocarbons for all ice cores.**

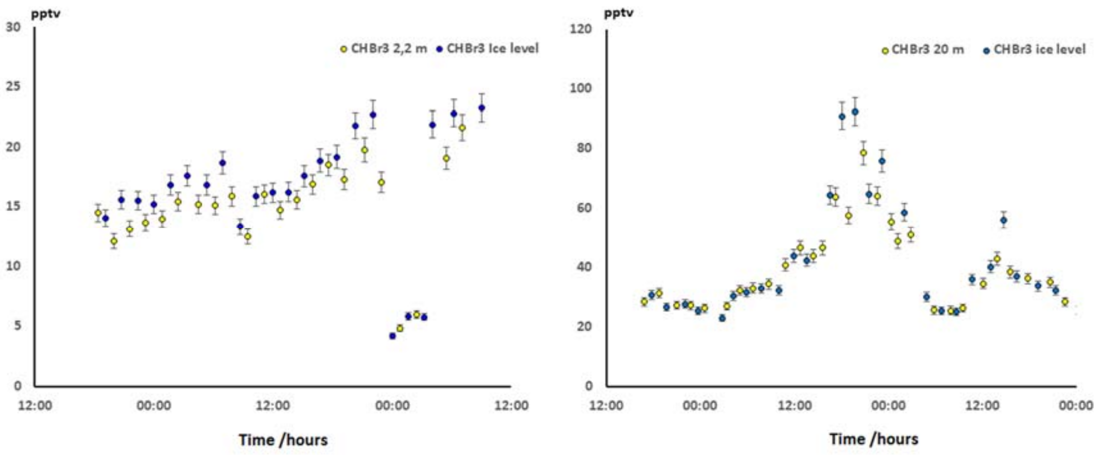

Supplementary Figure 3. Air gradient measurements of bromoform at stations 500 and 506. At station 500 (left graph) the samples were taken from the snow surface and 2,2 m above. At station 506 the samples were taken from snow surface and 20 m above. The instrumental errors are given as error bars.

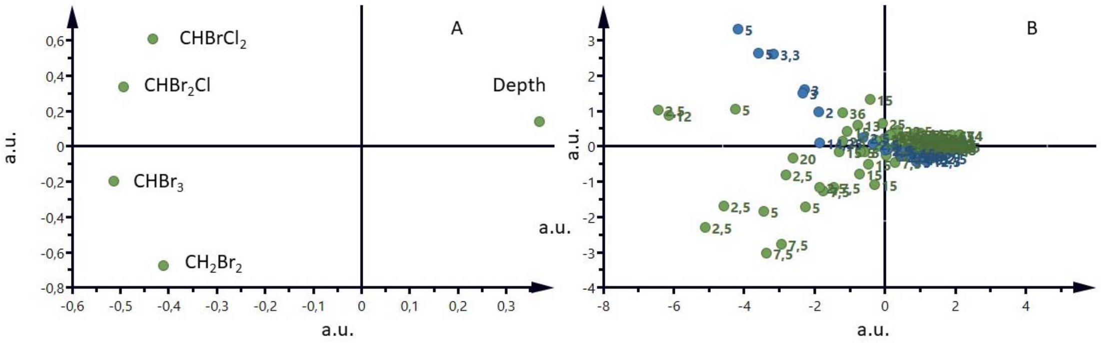

Supplementary Figure 4. Principal component analysis (PCA) of ice core profiles (green) and snow profiles (blue) of bromocarbon. The score scatter plot (A) shows the inverse relationship between the concentrations of bromocarbons and depth in ice cores and snow profiles. The first principal component (x-axis) describes the largest variation in the data set and the relationship between bromocarbons and depth in the ice cores. The loading plot (B) explains the score scatter plot. It can be seen that the highest concentrations of bromocarbons were found in the upper most part of the core and in the snow closest to the ice as indicated by the numbers (cm). All units are arbitrary units (a.u.).

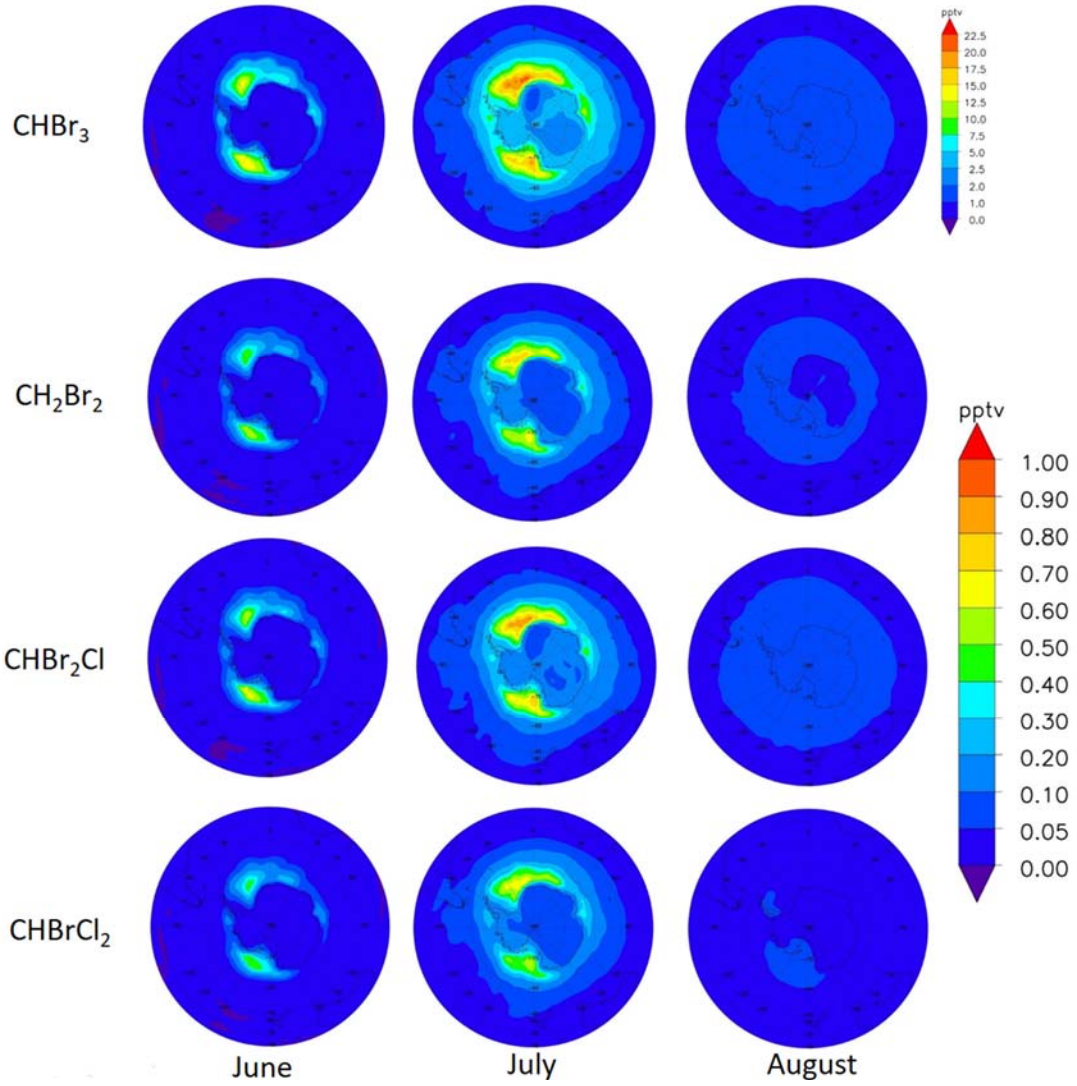

Supplementary Figure 5. **Modelled atmospheric distribution of bromocarbons emitted during Antarctic winter**. Geographical distributions of monthly-averaged surface air bromoform levels.

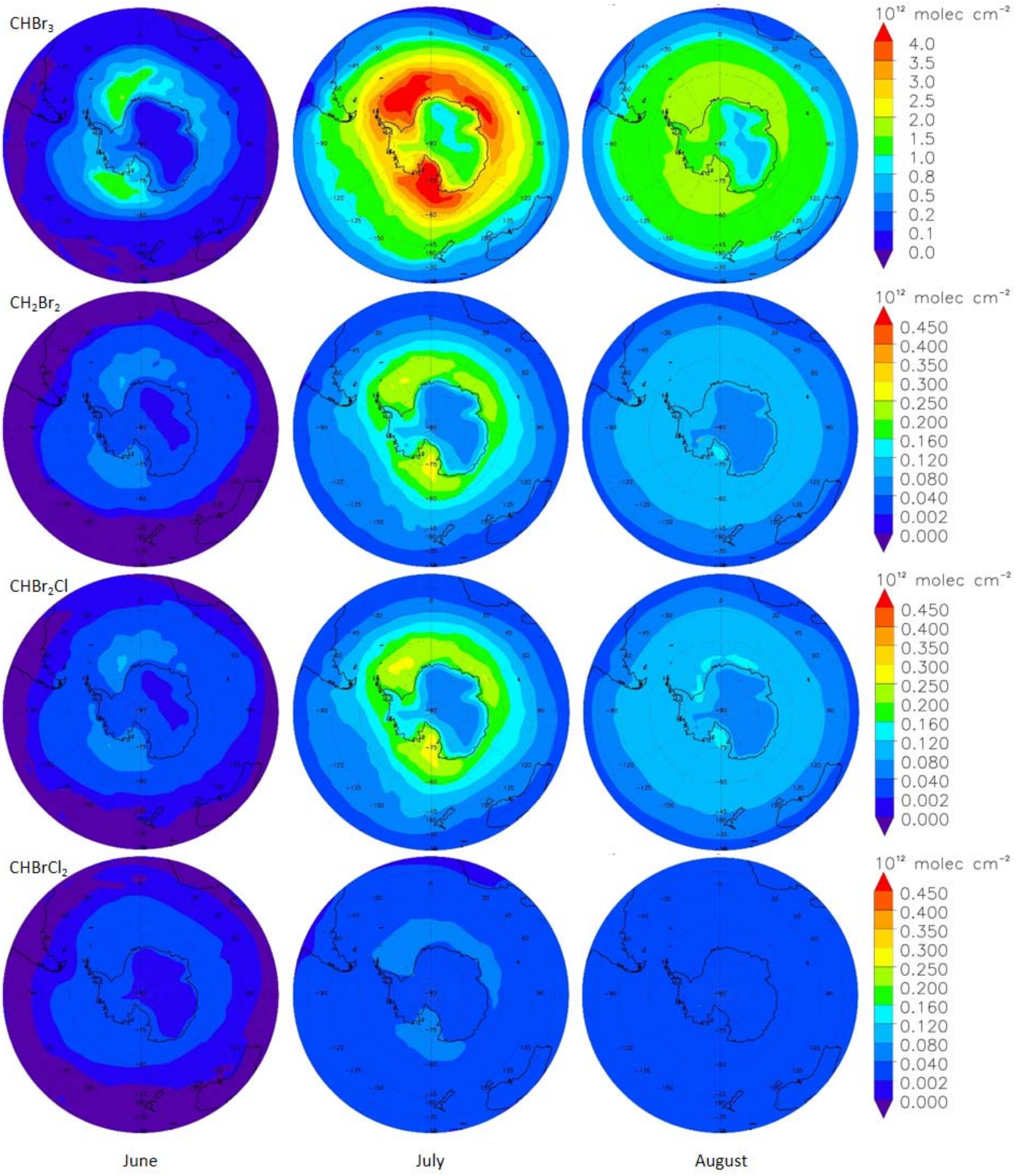

Supplementary Figure 6. Modelled distribution of monthly averaged tropospheric ($CHBr_3$, CH_2Br_2, $CHBr_2Cl$ and $CHBrCl_2$) over the Southern Hemisphere for the lower limit emission fluxes of bromocarbons. This spatial distribution is the result of sea ice emissions of bromocarbons during the Antarctic winter using the lower limit emission fluxes of bromocarbons from Table 1 (Flux in stations with Snow).

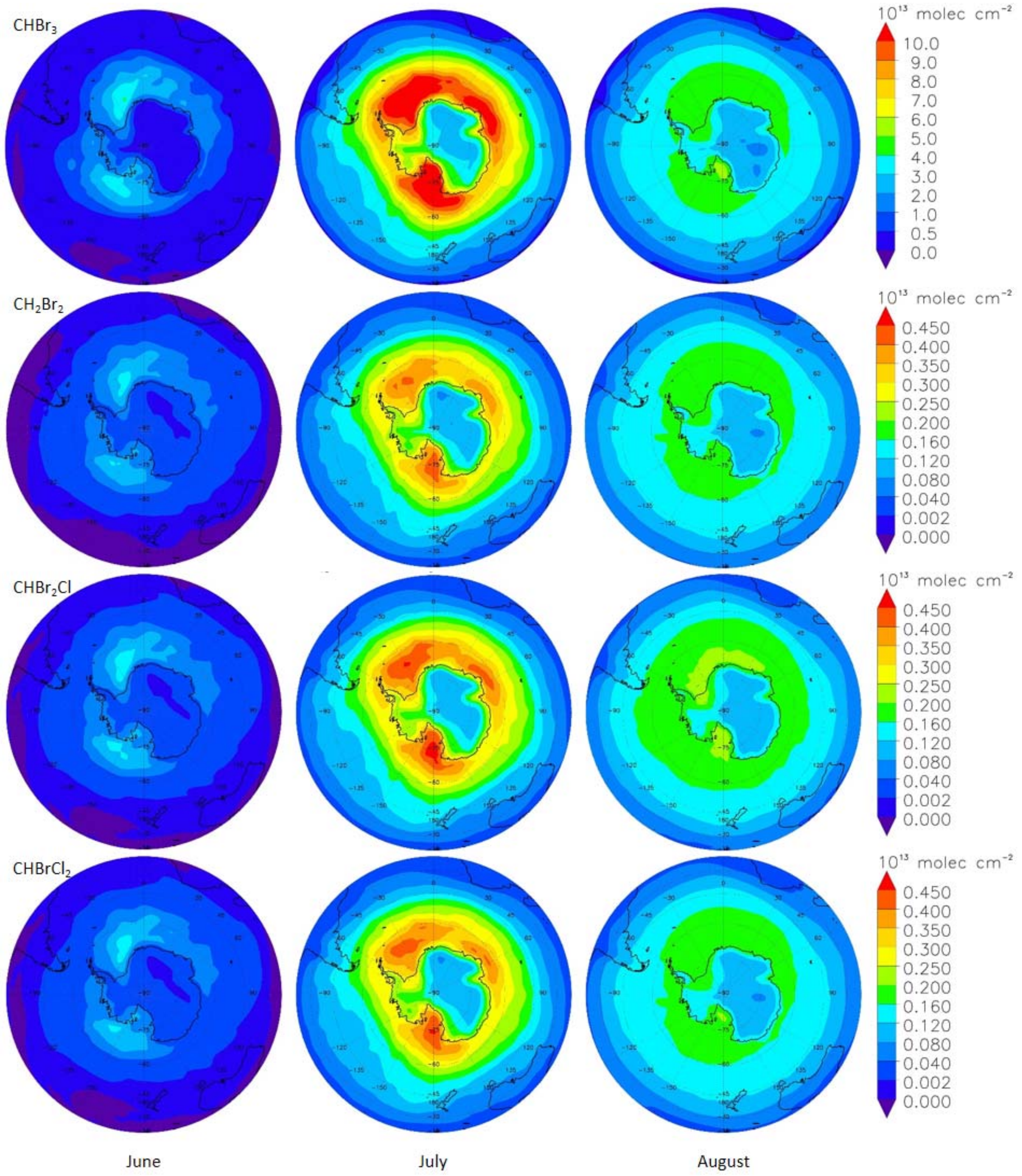

Supplementary Figure 7. Modelled distribution of monthly averaged tropospheric ($CHBr_3$, CH_2Br_2, $CHBr_2Cl$ and $CHBrCl_2$) over the Southern Hemisphere for the upper limit emission fluxes of bromocarbons. This spatial distribution is the result of sea ice emissions of bromocarbons during the Antarctic winter using the upper limit emission fluxes of bromocarbons from Table 1 (Flux in stations with Snow).

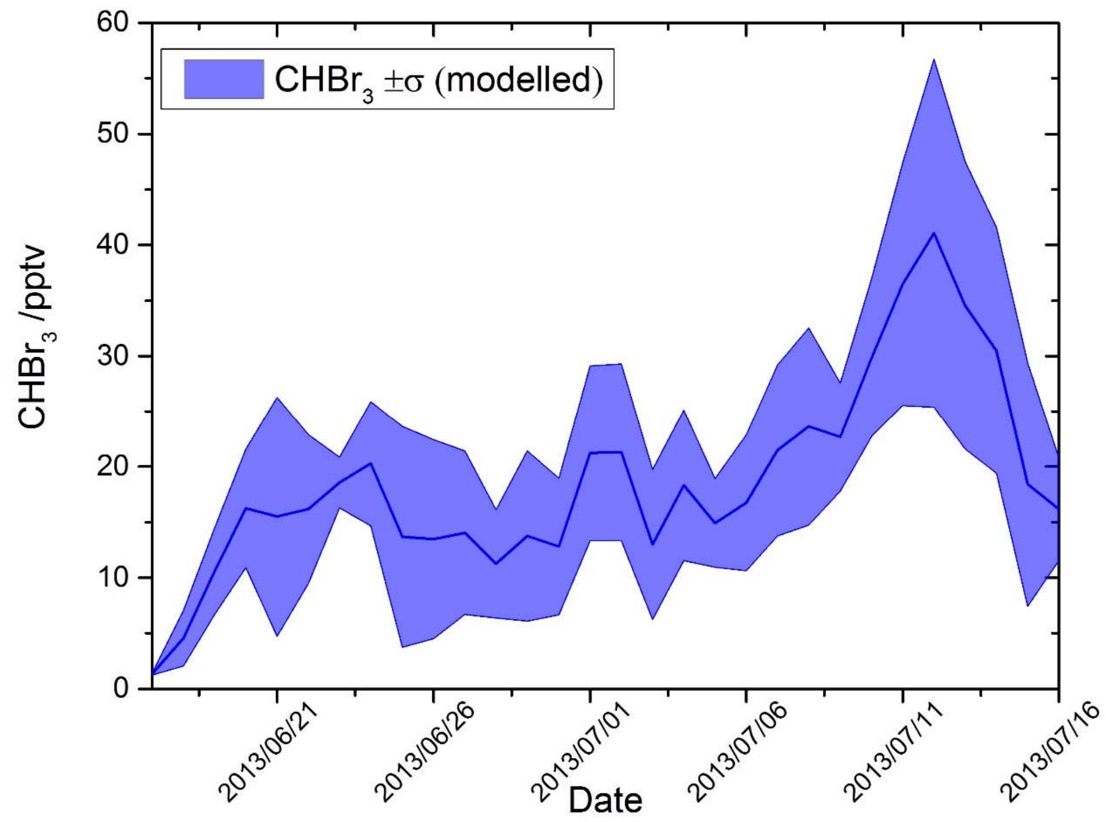

Supplementary Figure 8. **Modelled concentrations of bromoform ($CHBr_3$) in the measurements region.** Daily evolution of bromoform averaged over the zone in which the measurements were performed (latitude= 61.5-67.3ºS, longitude=0-23.3ºW).

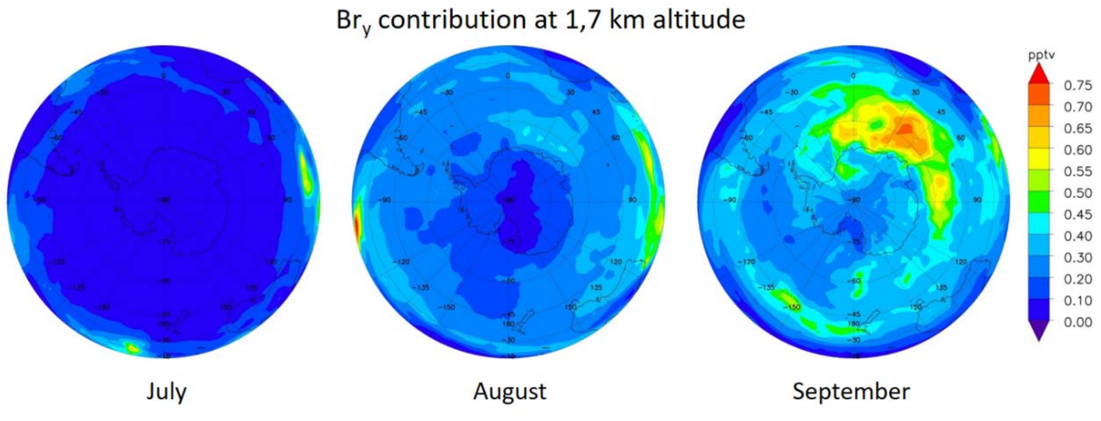

Supplementary Figure 9. Modelled mixing ratios of total reactive bromine (Br_y) in the lower free troposphere.

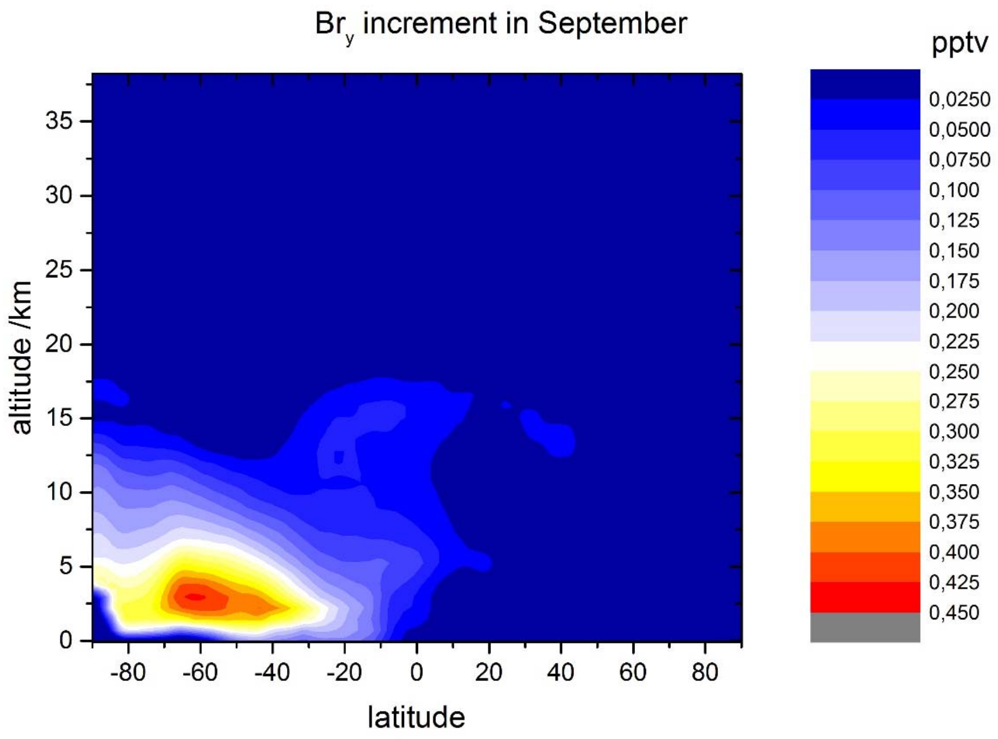

Supplementary Figure 10. **Latitudinal average of modelled mixing ratios of total reactive bromine in September**.

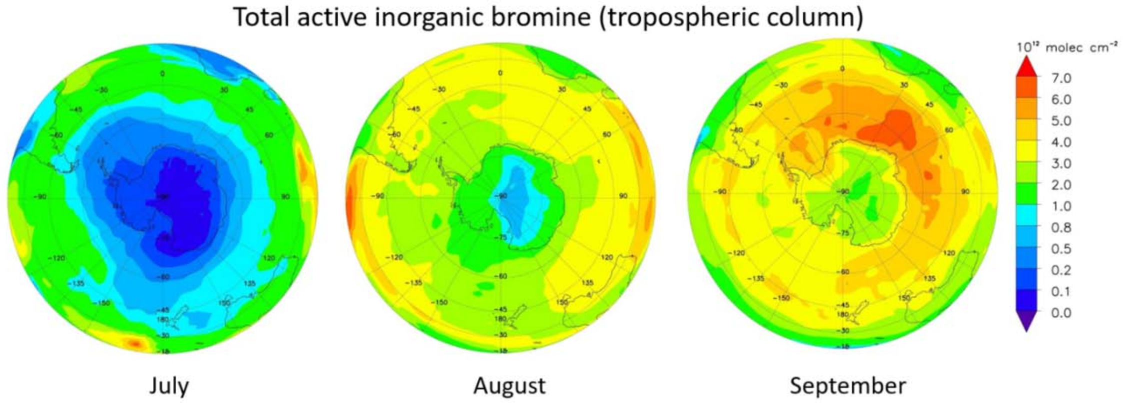

Supplementary Figure 11. **Total active inorganic bromine**. Monthly averages of the total tropospheric inorganic bromine resulting from the photochemical degradation of bromocarbons emitted from the Antarctic winter sea ice.

Supplementary Table 1. **List of sampling stations ANTXXIX/6**

Station	Date (yyyy- mm-	Latitude (°S)	Longitude (°W)	Ice core thickness [a] (cm)	Snow depth (cm)	Number of ice cores	Ice type[c]
486	2013-06-17	61.52	0.2	5	0	1	PI
488	2013-06-18	62.91	0.02	35	0	1	SI
489	2013-06-19	63.88	0.1	38	0-2	1	SI
493	2013-06-21	66.43	0.2	69-73.5	18.5	2	SI
496	2013-06-24	67.45	0.02	47-78	5	2	SI
497	2013-06-26	68.06	0.3	62[b]	30-34	1	SI
500	2013-07-03-2013-07-05	67.82	7.0	84-110	30.5	4	SI
503	2013-07-08	67.2	13.3	62-78	24.5	3	SI
506	2013-07-11-2013-07-14	67.36	23.3	47-67	6-20	8	SI

[a]Ice thickness = length of ice cores sampled for halocarbon analysis.
[b]Not whole ice thickness, could not retrieve whole ice core.
[c] SI = seasonal sea ice; PI = pancake ice

Supplementary Table 2. **Concentration ranges and median values for $CHBr_3$ and CH_2Br_2 in all ice cores. The values for sea ice temperature, salinity and calculated brine volume are also given.**

Station	Ice core depth (cm)	Range $CHBr_3$ (pM)	Median $CHBr_3$ (pM)	Range CH_2Br_2 (pM)	Median CH_2Br_2 (pM)	Temperature (^oC)	Bulk salinity (psu)	Brine volume (%)	No of samples
486	5	17-24	23	6,3 – 7,4	7,2	-5,3	19	20	3
488	4,5	740		22		m.v.	12	7	1
	13,5	92		13		m.v.	6	40	1
	22	41		7,7		m.v.	5,3	5	1
	31	210		7,5		m.v.	6,7	10	1
489	4	6300		160		m.v.	12	5	1
	11	180		29		m.v.	3,9	3	1
	20	51		8,8		m.v.	5	6	1
	32	66		14		m.v.	6	10	1
493	2,5	7000		1200		-11,7	8,2	4	1
	11	570		400		-12,2	6,6	3	1
	27	380		210		-11,8	6,1	3	1
	37	88		140		-10,7	5,2	2	1
	47	110		75		-9,49	4,4	2	1
	57	110		45		-9,05	5,3	3	1
	67	46		21		-6,85	6,1	4	1
496	5	280, 400		11, 22		-7,04	12	9	2
	15	49, 38		11, 12		-6,27	9,5	7	2
	25	59, 33		10, 12		-5,47	9	8	2
	35	34, 28		8,1, 7,9		-4,33	8	9	2
	43,5	43, 25		5,4, 5,3		-2,98	7,9	10	2
497	4	18000		390		-5,08	2,7	2	1
	12	3600		260		-5,72	4,3	4	1
	20	1500		260		-5,64	5,4	5	1
	28	1030		130		-5,83	7,1	6	1
	36	950		89		-6,13	6,6	5	1
	44	300		m.v		-5,99	8,9	7	1
	52	110		21		-5,69	10	8	1
	60	100		18		-5,98	7,2	6	1
500	5	74 - 1150	670	12 -210	140	-1,92 - -4,07	4,6 – 15	10 - 20	4

	15	60 - 1200	240	13 – 280	99	-2 - -4,31	3,6 – 7,9	6 – 9	4
	25	84 -760	310	21 – 250	110	-3,18 – -4,2	4,5 - 12	6 - 20	4
	35	44 - 390	220	23- 140	110	- 3,62 – -4,05	4,2 – 7,1	5 – 9	4
	45	17 - 200	130	22 – 110	66	-3,05 – -3,68	4,4 - 6	6 – 9	4
	55	19 - 170	110	30 – 91	35	- 3,18 – -3,44	5,7 - 6	8 – 8	4
	65	18 - 240	150	20 – 60	41	-3,03 - -4,05	5,2 - 8	6 - 10	4
	75	25 - 250	130	17 – 51	25	-2,81 - -3,18	4 - 7	5 - 10	4
	85	56 - 380	67	9,3 – 130	15	-2 - -4	4,2 – 6,2	7 – 9,7	4
	95	42 - 960	50	7,5 180	11	-1,92 - -3,62	4,6 – 10	10	3
	105	290		30		-2,3	7,5	15	1
503	5	340, 7900		690, 820		-8,08	8	5	2
	15	m.v, 480		m.v, 74		-7,55	6,3	4	2
	25	97, 150		40, 30		-6,68	6,8	5	2
	35	58, 96		29, 18		-5,62	8,1	7	2
	45	74, 52		20, 14		-4,75	6,4	6	2
	55	89, 34		17, 12		-3,95	4,3	5	2
	65	27, 29		8,5, 7,7		-2,9	3,6	6	2
	74	36, 25		6,3, 8,1		-2,46	3,8	7	2
506	2,5	970 - 3300	2100	180 – 510	280	-5,1 – 7,05	5 - 14	4 – 9	8
	7,5	130-1700	1500	80 – 490	240	-5,23 – -6,02	5,9 – 9,1	5 -7	6
	15	51 - 950	480	37 – 130	65	-4,09 - -5,39	3,6 – 6,2	4 – 7	8
	25	37 - 950	120	8,3 – 31	14	-3,13 - -4,44	3,3 – 5,3	4 - 7	8
	35	32 - 130	78	7,7 – 12	8,9	- 2,78 – -3,32	3,9 – 6,5	5 - 10	8
	45	19 - 82	45	3,9 – 8,3	7,1	-2,1 - -2,66	4 – 8,6	7 – 17	8

m.v.: missing value

Supplementary Table 3. Concentration ranges and median values for $CHBr_2Cl$ and $CHBrCl_2$ in all ice cores. The values for sea ice temperature, salinity and calculated brine volume are also given.

Station	Ice core depth (cm)	Range $CHBr_2Cl$ (pM)	Median $CHBr_2Cl$ (pM)	Range $CHBrCl_2$ (pM)	Median $CHBrCl_2$ (pM)	No of samples
486	5	1,9 – 2,5	2,3	1,6 -1,9	1,8	3
488	4,5	44		7,2		1
	13,5	9,9		3,8		1
	22	5,9		3,1		1
	31	11		2,3		1
489	4	250		26		1
	11	14		18		1
	20	6,5		6,2		1
	32	4,6		2,4		1
493	2,5	250		76		1
	11	44		24		1
	27	26		14		1
	37	20		16		1
	47	21		24		1
	57	22		20		1
	67	11		11		1
496	5	15, 26		13, 45		2
	15	4,7 – 4,9		8,5, 8,9		2
	25	4,7 – 5,3		7,9, 18		2
	35	4,7 – 4,1		12, 13		2
	43,5	3,3 – 4,2		4,5, 10		2
497	4	1100		110		1
	12	160		23		1
	20	75		13		1
	28	56		10		1
	36	65		14		1
	44	20		4,5		1
	52	7,4		2,6		1
	60	7,6		3,0		1

500	5	9,9 – 24	16	2,6 -5,2	4,9	4
	15	7,7 – 25	10	2,7 -7,8	3,8	4
	25	6,6 – 18	9,9	2,8 – 5,6	3,6	4
	35	6,3 – 100	11	2,3 – 270	4,7	4
	45	5,4 – 9,7	6,8	2,9 – 3,1	3,2	4
	55	5,9 – 7,5	6,6	3,1 – 5,2	3,3	4
	65	4,8 – 10	6,4	2,6 – 4,8	3,2	4
	75	6,0 – 9,9	8,0	2,8 -5,1	3,4	4
	85	2,6 – 12	6,9	2,3 – 4,6	2,9	4
	95	3,9 - 22	5,8	2,2 – 4,5	3,7	3
	105	8,5		1,8		1
503	5	47, 120		10, 19		2
	15	m.v., 15		m.v., 6,6		2
	25	6,1, 8,3		3,2, 6,1		2
	35	4,5, 5,9		2,5, 3,8		2
	45	4,5, 6,6		2,7, 4,7		2
	55	6,3, 5,3		3,9, 3,9		2
	65	4,4, 5,1		3,8, 3,9		2
	74	4,9, 4,0		3,2, 3,1		2
506	2,5	39 – 150	87	6,9 – 37	11	8
	7,5	8,7 – 86	41	6,3 – 7,9	6,3	6
	15	10 – 63	18	3,5 -20	7,2	8
	25	5,0 – 18	11	3,9 – 7,4	4,9	8
	35	3,0 – 10	4,5	2,0 – 4,1	2,4	8
	45	2,2 – 5,0	3,5	1,4 -3,4	1,9	8

Supplementary Table 4. **Snow concentrations and median values of bromocarbons together with snow salinity**. The depths are given as cm from ice surface.

Station	Snow depth (cm)	Range $CHBr_3$(pM)	Median $CHBr_3$ (pM)	Range CH_2Br_2 (pM)	Median CH_2Br_2 (pM)	Range $CHBr_2Cl$ (pM)	Median $CHBr_2Cl$ (pM)	Range $CHBrCl_2$ (pM)	Median $CHBrCl_2$ (pM)	Salinity (psu)	No of samples
486	no snow										
488	no snow										
489	no snow										
493	13	74		1,5		3,3		0,49		0,1	1
	4	1600		38		60		4,6		5,5	1
496	2,5	60 - 210	140	0,35 – 4,0	1,1	2,5 - 11	6,9	0,42 – 1,4	0,95	5,2 – 9,5	6
497	29	8,6		0,36		0,51		0,14		0	1
	19	94		1,6		4,5		,67		0,7	1
	9	170		3,0		7,7		0,92		0,9	1
	2	3050		53		210		28		3,8	1
500	35	1,8, 1,5		0,12, 0,13		0,15, 0,24		0,04, 0,13		0,5, 0	2
	25	2,0, 4,0		0,09, 0,17		0,24, 0,21		0,07, 0,08		0, 0,2	2
	10	11,120		0,55, 3,3		0,53, 5,1		0,10, 0,72		4,6, 16	2
	3	120		19		6,9		1,3		24	1
503	20	11		0,22		0,55		0,12		0	1
	10	73		0,96		4,9		0,68		0,3	1
	3	560		10		31		9,1		4,1	1
506	12	3,6 - 80	31	0,13 – 1,1	0,54	0,3 – 7,1	4,1	0,07 – 1,0	0,50	0 – 8,5	4
	3	640 - 1400	910	9,7 - 19	13	48 - 140	85	9,6 - 24	18	5,9 - 9	4

Supplementary Table 5. Fluxes of bromocarbons from individual stations (nmol m^{-2} h^{-1}). Flux* is based on diffusion through snow and Flux** is based on sea-air flux model. The median values are given in the cases of multiple snow profiles or ice cores. The number of samples can be found in Supplementary Tables 2,3 and 4. The error of all fluxes included in this table is 13%, based on the measurement uncertainties in concentrations and in the snow depth measurements.

Station	Flux* $CHBr_3$	Flux ** $CHBr_3$	Flux* CH_2Br_2	Flux** CH_2Br_2	Flux * $CHBr_2Cl$	Flux** $CHBr_2Cl$	Flux * $CHBrCl_2$	Flux** $CHBrCl_2$	ice cores	snow profiles
488	n.s.	8000	n.s.	20	n.s.	50	n.s.	3	1	1
489	n.s.	17000	n.s.	50	n.s.	70	n.s.	5	1	1
493	100	1000	5	60	6	10	0.6	5	1	1
496	20	20; 40	0,6	1; 3	1	1; 3	0.6	1; 6	2	1
497	200	8000	200	1200	20	400	4	40	1	1
500	8	70	0.5	17	0.5	2	0.1	0,09; 0.3	4	1
503	40	3; 100	1	10; 20	3	1; 5	8		2	1
506	60	70	2	8	8	3	2	0.2	8	3

n.s. no snow cover

Supplementary Table 6. Limits of detection (pM) for brominated hydrocarbons.

Water	Velocity XPT	Custom built system I	Custom built system II
CH_2Br_2	0,031	0,014	0,022
CH_2BrCl_2	0,031	0,018	0,048
$CHBr_2Cl$	0,029	0,036	0,055
$CHBr_3$	0,15	0,10	0,14
Air			
CH_2Br_2		0,001	
CH_2BrCl_2		0,002	
$CHBr_2Cl$		0,004	
$CHBr_3$		0,08	
