# Supplementary material for: Organic bromine compounds produced in sea ice in Antarctic winter
Source: Nat Commun. 2018 Dec 11;9:5291. doi: 10.1038/s41467-018-07062-8 (PMC6290016; doi:10.1038/s41467-018-07062-8)
Supplement: Supplementary file 2 — Description of Additional Supplementary Files [file 41467_2018_7062_MOESM2_ESM.pdf]

Description of Additional Supplementary Files

File Name: Supplementary Data 1

Description: Supplementary data file in spreadsheet format (comma separated) containing summary details and details of the timing of events during 48 recorded dives, normalized to the time at which the male is closest to the female in the dive. These data are used to produce the main results in Figure 2. Columns describe (in order); the maximal speed (m s^{-1}) reached within each dive ("MaxSpeed"), the time at which the male takes up the greatest degree of female visual angle ("TMaxVisAngle"), the time at which the male reaches maximal speed ("TMaxSpeed"), the time at which the male reaches maximal horizontal velocity ("TMaxXVel"), the time at which the male reaches maximal vertical velocity ("TMaxYVel"), the time at which sonations begin ("TSonStart"), the time at which sonations end ("TSonEnd"), the time at which the gorget is estimated to become visible (TGorgetVisible"), the time at which the gorget is estimated to become hidden ("TGorgetHidden"), the time at which the gorget is predicted to be most bright ("TMaxLum"), the time at which the gorget is predicted to most stimulate the long-wavelength sensitive cone of the female ("TMaxLWS"), the time at which the gorget is predicted to change in long-wavelength stimulation at the greatest rate ("TMaxColShift"), the maximal estimated upward Doppler shift predicted during the dive ("MaxDoppler"), the maximal estimated downward Doppler shift predicted during the dive ("MinDoppler"), which video recording/bout of diving the dive came from ("bout").
